# Detection of HBV Genotypes of Tumor Tissues and Serum by A Fluorescence Polarization Assay in North-Western China's Hepatocellular Carcinoma Patients

**DOI:** 10.1186/1743-422X-8-362

**Published:** 2011-07-22

**Authors:** Jianguo Lu, Weidong Gong, Hong Cheng, Zhiqun Wu, Ding Li, Xiangling Wang, Ping Liang, Ju Zhang

**Affiliations:** 1 Department of General Surgery, Tangdu Hospital, The Fourth Military Medical University, Xian, China; 2 Department of Interventional radiology, Tangdu Hospital, The Fourth Military Medical University; Xian, China; 3 Department of Pathology, Xijing Hospital, The Fourth Military Medical University, Xian, China; 4 Center of Biotechnological Diagnosis and Therapy, the 261st Hospital of PLA, Beijing, China; 5 Department of Clinical Laboratory, Xian Jiaotong University, Xian, China; 6 State Key Laboratory Of Cancer Biology, Institute of Gene Diagnosis, School of Pharmacy, The Fourth Military Medical University, 169 Changle West Road, Xian, Shaanxi, 710032, China

**Keywords:** hepatitis B virus genotype, hepatocellular carcinoma, fluorescence polarization, north-western China

## Abstract

**Background:**

The understanding of the distribution of hepatitis B virus genotypes and the occult hepatitis B virus infection in hepatocellular carcinoma may shed light into the prevention and treatment of hepatocellular carcinoma. The purpose of the study is to investigate hepatitis B virus genotypes distribution, the high-risk genotypes and the occult infection in north-western China's hepatocellular carcinoma patients.

**Methods:**

Hepatitis B virus genotypes A-D of hepatocellular carcinoma tumor tissues and serum samples in 268 north-western China hepatocellular carcinoma patients were detected by fluorescence polarization assay. The hepatitis B virus genotypes in serum and matched primary tumor tissue samples were compared. Hepatitis B surface antigen and α-fetoprotein in serum were detected. Occult hepatitis B virus infections were analyzed. The relationship between hepatitis B virus genotypes and clinicopathologic characteristics were analyzed statistically using SPSS v.10.0.

**Results:**

Intrahepatic hepatitis B virus DNA was detected in 83.6% of 268 patients, whereas serum hepatitis B virus DNA was detected in 78.7%. The hepatitis B virus genotypes in serum were consistent with the results in matched tumor tissue. Intrahepatic hepatitis B virus genotype B and C were detected respectively in 11.6% and 54.5% of the patients. Mixed intrahepatic hepatitis B virus genotypes were detected in 13.4% of 268 patients. There was not mixed hepatitis B virus infection in Edmondonson grade I. The patients with mixed HBV genotypes exhibited statistically significant different Edmondson grade than the patients with single type HBV infection (p < 0.05). Hepatitis B surface antigens were positive in 77.2% of 268 patients. Hepatitis B virus genotype C was detected in 64.7% of occult infected patients. There was no significant differences of patients' ages and α-fetoprotein level in different groups of intrahepatic hepatitis B virus genotypes (p > 0.05).

**Conclusions:**

Hepatitis B virus genotype C was associated closely with the development of hepatocellular carcinoma and the occult hepatitis B virus infection in patients in north-western China. There was a relatively high prevalence of mixed hepatitis B virus infection in Edmondonson grade III-IV.

## Background

Hepatocellular carcinoma (HCC) is one of the most common malignant tumors worldwide. Hepatitis B virus (HBV) infection is strongly associated with the occurrence and development of HCC [1]. HBV can be classified into eight genotypes (designated by capital letters A-H) based on an inter-group divergence of 8% or more in the complete nucleotide sequence and HBV genotypes affect the clinical course of HCC and response to treatment. HBV genotypes have a pattern of geographical distribution. The HBV genotype A, B, C and D has been found in China [2,3]. The understanding of the distribution of HBV genotypes and the occult HBV infections in HCC may shed light into the future prevention and treatment of HBV-related HCC in China.

Advances in molecular biotechnology have allowed the detailed study of the viral genotypes of HBV and the occult HBV infections. Numerous studies have been done on investigating the distribution and the impact of HBV genotypes in HCC. HBV genotype C has been found to be a higher risk factor for development of HCC as compared with HBV genotype B in Taiwan [4]. However, the distribution of HBV genotypes, the high-risk genotypes of HBV and the occult HBV infections in HCC have not been investigated in north-western region of China. In this study, HBV genotypes A-D of primary tumor tissues and serum samples in 268 north-western China HCC patients were detected by a simple and cost-effective fluorescence polarization (FP) assay and the occult HBV infections were investigated [5].

## Materials and methods

### Patient population, samples and DNA extraction

From January 2008 to June 2010, 268 patients with pathologically confirmed HCC, everage age of 54.75 ± 11.69 (interquartile range, 29-76) years, 218 male patients and 50 female patients, who underwent surgical resection or hepatic puncture in Tangdu Hospital and Xijing Hospital of the Fourth Military Medical University, and Xian Jiaotong University, Xian, China, were included in this study. Primary tumor tissue sample was surgically obtained or hepatic puncture from each patient. Areas of tumor tissue were previously delineated for each sample by microscopic examination of a reference slide stained with H&E. Histological examinations were carried out and the final diagnosis was made by pathologists. Matched tumor tissue and serum samples were obtained from all the HCC patients. All the patients who had not received hepatitis B vaccination signed informed consent to participate in this study and gave permission for the use of their serum and tumor tissues samples. All the samples were stored in liquid nitrogen until use. The study was in compliance with the Helsinki Declaration and was approved by the Human Research Protective Committee of The Fourth Military Medical University (CNTG0801).

Hepatitis B surface antigen (HBsAg) in serum was detected with ELISA assays (Kehua, Shanghai, China) and α-fetoprotein (AFP) in serum was detected with Elecsys AFP Roche Diagnostics GmbH (Roche, Switzerland). The patients' clinical and pathological characteristics were reported in Table 1.

**Table 1:** Intrahepatic HBV-genotypes and Clinicopathologic Characteristics

	Total	Grade I	Grade II	Grade III - IV	HBsAg	AFP(ng/L)	Age
No.(%)	268	85(31.7%)	123(45.9%)	60(22.4%)	Positive	(X ± SD)	(X ± SD)
HBV DNA							
Positive	224	83	102	39	207	359 ± 164	54.73 ± 10.7
HBV A	1	0	1	0	0	338 ± 54	50.92 ± 6.3
HBV B	31	13	14	4	27	407 ± 163	51.71 ± 12.2
HBV C	146	69	64	13	135	368 ± 191	56.27 ± 9.3
HBV D	10	1	5	4	9	433 ± 151	54.21 ± 7.2
Mixed	36	0	18	18	36	387 ± 179	53.98 ± 11.6
HBV DNA							
Negative	44	2	21	21	0	374 ± 160	58.46 ± 14.3

Genomic DNA was extracted respectively from the matched serum and primary tumor tissue. DNA extracted from tumor tissue by the DNeasy Tissue Kit (Qiagen, Hilden, Germany) according to the manufacturer's instructions and eluted in Tris-EDTA buffer (10 mM Tris-HCl [pH 8.0] and 1 mM EDTA disodiumsalt), stored at -70°C. DNA extracted from serum by the QIAamp DNA Blood Mini kit (QIAGEN, Hilden, Germany) following the manufacturer's instructions and eluted in Tris-EDTA buffer, stored at -70°C.

All the tumor tissue samples were checked by PCR assay using primers specific for α-tubulin to exclude the presence of inhibitors and to assess the quality and the integrity of the extracted DNA as described previously [6]. All the primers and probes were synthesized and labeled by Invitrogen (Shanghai, China).

### HBV DNA amplification by a single asymmetric PCR

In this study, we investigated the presence of HBV DNA genotypes A-D by the FP assay based on a single asymmetric PCR and hybridization according to the previously described method. The minimum level of the FP assay was 2.5 copies and the FP assay was able to detect the minor population of HBV genotypes even when their contents were as low as 1% [5]. HBV negative serum containing pGEM-T-HBV-A, pGEM-T-HBV-B, pGEM-T-HBV-C, pGEM-T-HBV-D were amplified as positive controls. HBV negative serum containing pGEM-T-easy Vector DNA was amplified as negative controls. The DNA extracted from serum and tumor tissue of each sample with successful amplification of α-tubulin was subjected to the asymmetric PCR. Each sample was analyzed in duplicate. A pair of general primers (HBV-FOR 5'-tcaccatattcttgggaacaaga-3'and HBV-REV 5'-ttcctgaactggagccacca-3'), with a high sensitivity for the amplification of HBV genotypes A to D, were used in this study. In brief, 5 μl of the DNA extraction from each sample was subjected to an asymmetric PCR. An asymmetric PCR was carried out in 25 ul PCR reaction buffer containing 1.25 U Taq polymerase (Promega, USA), 0.2 mM dNTP (each), unequal amounts of the two primers (10 pmol HBV-FOR and 80 pmol HBV-REV primers). Finally, the reaction mixture was subjected to an additional cycle without extension so as to generate mainly single-stranded DNA.

### Detection of HBV-genotypes by the fluorescence polarization assay

The determination of genotypes A-D of HBV was done simultaneously by the FP assay with four labeled genotype-specific probes (HBV-A-probe, HBV-B-probe, HBV-C-probe and HBV-D-probe), according to the previously described method with modifications [5]. The four probes hybridized with one of the strands of the target amplicons were labeled with different fluorescent reporter dye (TAMRA, FAM, ROX, BTR) to differentiate the amplification of every HBV genotype. The asymmetric PCR products generated from every sample were subjected to the FP assay. In brief, 200 ul mixtures of the four probes and 20 ul of the asymmetric PCR products were incubated at 48°C for 30 min. The FP values of reaction buffer were measured using the Fluorescence Polarization Capable Instrument (YG-06, Yangguang, Shanxi, China) and the HBV genotypes were determined by the increased FP values. The reaction was performed in duplicate.

## Results

### HBV genotypes in serum and primary tumor tissue samples

In this study the presence of HBV DNA genotypes in primary HCC tumor tissue and serum samples were detected by the FP assay (Table 1). Intrahepatic HBV DNA was detected in 83.6% (224/268) of the HCC patients, whereas intrahepatic HBV DNA was not detected in 16.4% (44/268) of the HCC patients. Meanwhile, HBV-DNA in matched serum samples was only detected in 79.1% (211/268) of the HCC patients. The HBV DNA-genotypes in serum from the 211 patients were consistent with the results in the matched primary tumor tissue.

**Figure 1:**
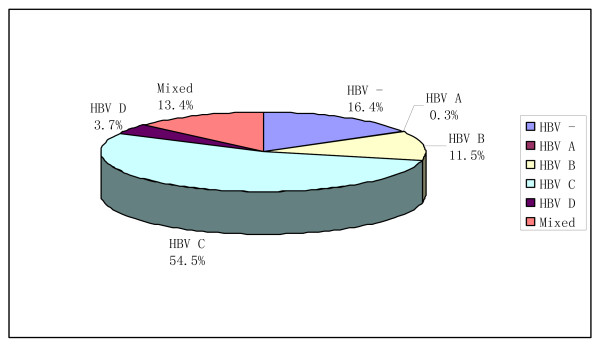
**Distribution of HBV genotypes in HCC tissues**.

Among intrahepatic HBV DNA positive samples, mixed HBV DNA-genotypes were detected in 13.4% (36/268) of the HCC patients, 28 cases with HBV-genotype B and HBV-genotype C and 8 case with HBV-genotype C and HBV-genotype D. HBV-genotype C(54.5%) and B(11.5%) attributed predominantly to intrahepatic HBV infections in the HCC patients, whereas HBV genotypes A infection (0.3%) was the least observed. Distribution of HBV genotypes in HCC tissues was shown in Figure 1.

### Occult HBV infection of the HCC patients

**Figure 2:**
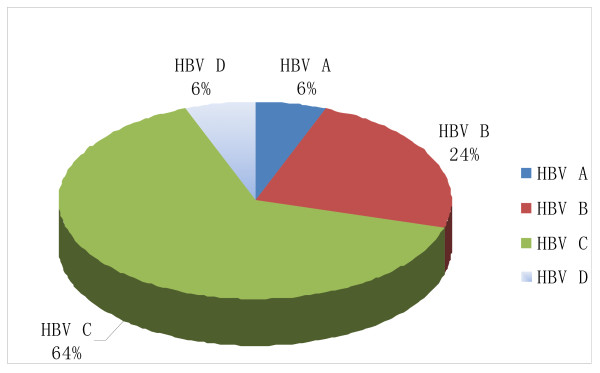
**Distribution of HBV genotypes in HCC tissues with occult HBV infection**.

Occult HBV infection is defined by the presence of HBV DNA in serum or liver tissue in the absence of HBsAg [7]. In this study, HBsAg were positive in only 77.2% (207/268) of the HCC patients. Occult HBV infections were found in 17 HCC patients. Four HCC patients with both serum and intrahepatic HBV-genotype C positive were detected with HBsAg negative. Thirteen HCC patients with intrahepatic HBV-DNA positive but serum HBV-DNA negative were detected with HBsAg negative. No mixed intrahepatic HBV infection was found in the 17 occult HBV infection HCC patients. Distribution of HBV genotypes in HCC tissues with occult HBV infection was shown in Figure 2.

### Relation between intrahepatic HBV DNA-genotypes and clinicopathologic characteristics

High AFP level in serum is considered as an important clinical marker for the happening of HCC. Our study investigated the AFP level in the HCC patients. The potential relationship of intrahepatic HBV-genotypes, AFP level and the patients' ages were analyzed using SPSS v.10.0 software (SPSS Inc., USA). There were no statistically significant differences of the patients' ages and AFP level in different groups of intrahepatic HBV-genotypes (Table 1). The prevalence of occult HBV infection did not differ with patients' ages and AFP level.

The potential relationship between intrahepatic HBV-genotypes and the tumor Edmondonson grades were also being investigated. No mixed HBV infection was found in the patients with Edmondson grade I. There was a higher percentage of mixed HBV infection (18/60) in Edmondonson grade III-IV than that (18/123) in Edmondonson grade II. A higher percentage of patients with mixed HBV genotypes exhibited a more severe HCC based on Edmondson grade, this was statistically significant different with patients of single type HBV infection (p < 0.05).

## Discussion

HBV DNA was frequently found positive in HCC patients. HBVs are able to persist in the host for years and the continuous intrahepatic inflammation maintains a cycle of liver cell destruction and regeneration that often results in HCC [8]. The incidence of HCC is high in the world, and 43% of the HCC patients reside in China. HBV genotypes affect the clinical course of infection and development of HCC [9]. It has been reported that HBV genotype C and D were associated with an increased risk of HCC compared with other HBV genotypes, HBV genotype C and D were associated with more severe liver diseases and rapid fibrosis development [10]. And HCC patients with HBV genotype C have a greater tumor recurrence rate compared with those with HBV genotype B [11]. The differences in the immunopathogenesis between various HBV genotypes have been reported [12]. Detection of the HBV genotypes in HCC would be useful for the study of the epidemiology and molecular mechanism of HBV in the development of HCC. The distribution of HBV genotypes in HCC varies in different countries and even within different regions of the same country [13]. In mainland China, HBV genotype C is predominant in Beijing and Shandong and HBV genotype B in Hunan and Fujian; whereas HBV genotype B and HBV genotype C are almost equally prevalent in Yunnan and Guangdong [14]. The incidence of HBV-related HCC is high in north-western China. Considering that HBV genotypes A-D are predominantly in China, we detected HBV genotypes A-D by an improved FP assay in serum and matched primary tissue samples of the HCC patients in north-western China. The FP assay was simple and cost-effective because HBV genotypes A-D could be detected by a single PCR reaction [5]. This is the first study to show HBV genotypes in the HCC patients in north-western China. This study showed, as in Table 1, that intrahepatic HBV infections were attributed predominantly to HBV genotypes C and B, that constituted 65.2% and 13.8% respectively. In addition, there was a relatively high prevalence of mixed infections of 16.1% (36/224) in the studied group. HBV genotype C was found in all the mixed infections. This study suggested that HBV-genotype C infections were the most common causes of HCC in the patients in north-western China.

The diagnosis of HBV infection is usually based on the detection of HBsAg in blood. However, HBsAg seroclearance does not necessarily imply HBV DNA eradication and HBsAg negative were not sufficient markers to exclude HBV DNA carriers [15,16]. Occult HBV infection might occur after complete clinical recovery from acute self-limited hepatitis or because of HBV DNA mutation. HBV might be reactivated in patients with decreased immune status undergoing anticancer chemotherapy [17-20]. The persistent HBV infections have a critical role in the development of HCC in HBsAg-negative patients. Occult HBV infection might be associated with more severe liver damage, cryptogenic chronic hepatitis, more advanced fibrosis. Furthermore, occult HBV infection affect responsiveness to therapy and disease progress and the HBV genotypes influence the frequency of occults HBV[21-26]. The incidence of occult HBV infection is different in different countries. Despite the importance of the occult HBV infection, there is little information of HBV genotypes and its relation with occult infection in the HCC patients in north-western China. In this study, occult HBV infection and the genotypes of occult HBV infection in the HCC patients in north-western China were investigated. In the 268 HCC patients, occult intrahepatic HBV infection was detected in 17 cases. The occult intrahepatic HBV infections in the HCC patients were predominantly to genotypes C that constituted 64.7% (11/17). Our results revealed that occult HBV genotypes C infections were closely related with the occurrence of HCC in north-western China.

It is well recognized that serum is not the best source for the detection of HBV DNA in occult HBV infection and that the liver constitutes the most reliable source of material. This study suggested that the detection of HBV-DNA in primary tumor tissue has a higher sensitivity than that in matched serum. Nonetheless, it was documented that peripheral blood mononuclear cells (PBMC) also harbour HBV DNA and its replication intermediates and they should be considered when detection of low levels of HBV DNA is attempted [27,28]. HBV DNA was not detected in PBMC in this study. The limitations of the approach would be investigated in future.

## Conclusions

HBV genotypes distribution and occult HBV infection in the HCC patients in north-western China has been investigated. The study suggested that HBV genotype C infections were associated closely with the development of HCC and the occult HBV infection in the HCC patients in north-western China. There was a relatively high prevalence of mixed infections in Edmondonson grade III-IV in the studied group. This would be useful for the study of the epidemiology and molecular mechanism of HBV genotypes on HCC development and therapy in future.

## Competing interests

The authors declare that they have no competing interests.

## Authors' contributions

JL, WG and HC contributed equally to this work. All authors read and approved the final manuscript.
